# Carotenoids‐based reddish pelvic spines in nonreproducing female and male sticklebacks (*Gasterosteus aculeatus*) – Signalling social dominance?

**DOI:** 10.1002/ece3.7892

**Published:** 2021-07-08

**Authors:** Karl Kristian Kroken, Axel Aas Sæthre, Ove Nicolaisen, Torvald Blikra Egeland, Jarle Tryti Nordeide

**Affiliations:** ^1^ Faculty of Biosciences and Aquaculture Nord University Bodø Norway; ^2^ Faculty of Education and Arts Nord University Bodø Norway

**Keywords:** carotenoids, dominance, *Gasterosterus aculeatus*, ornament, pelvic spine, signal

## Abstract

Conspicuous ornaments are often considered a result of evolution by sexual selection. According to the social selection hypothesis, such conspicuous traits may also evolve as badges of status associated with increased boldness or aggression toward conspecifics in conflicts about ecological resources. This study tested predictions from the social selection hypothesis to explain evolution of conspicuous red color of the pelvic spines of the three‐spine stickleback (*Gasterosteus aculeatus*). Wild nonreproducing sticklebacks were presented to pairs of dummies which differed at their pelvic spines, having either (i) normal‐sized gray or red pelvic spines or (ii) normal‐sized gray or large red pelvic spines. The experimental tank was illuminated by white or green light, since green light impedes the sticklebacks’ ability to detect red color. The dummies moved slowly around in circles at each end of the experimental tank. We quantified the parameters (i) which of the two dummies was visited first, (ii) time taken before the first visit to a dummy, (iii) distribution of the focal sticklebacks in the two zones close to each of the two dummies and in the neutral zone of the tank, (iv) close to which of the two dummies did the focal fish eat its first food‐piece, and (v) time spent until the first piece of food was eaten. This was carried out for 22 females and 29 males sticklebacks. The results suggested no effect of the color or size of the dummies’ pelvic spines, on none of the five behavioral parameters. Moreover, neither the color of the pelvic spines of the focal sticklebacks themselves (as opposed to redness of the dummies’ spines) nor their body length was associated with behavior toward the dummies. Thus, this study did not support predictions from the social selection hypothesis to explain evolution of red pelvic spines in sticklebacks.

## Graphical abstract

The behavior of male and female three‐spine sticklebacks towards pair dummies was quantified in a controlled tank experiment. The dummies differed in the conspicuousness of their ornamented reddish pelvic spines. None of the quantifications of the behavioral of wild fish towards the two dummies nor the color of the pelvic spines of the live sticklebacks themselves was associated with the behaviour towards the dummies. This study did not support predictions from the social selection hypothesis to explain evolution of red pelvic spines in sticklebacks.

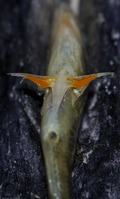

## 1 INTRODUCTION

Extravagant ornaments and weaponry have evolved because they increase individuals’ sexual attractiveness (Darwin, 1871). Darwin's ideas about sexual selection developed mainly based on his thinking of the evolution of ornaments in the more conspicuously decorated males, although he recognized evolution of ornaments in both sexes. The growing popularity in evolution of female ornaments the last couple of decades has mainly focused on testing three hypotheses to explain ornaments in species with conventional sex roles. Firstly, according to the “direct selection” hypothesis, both female‐specific and mutual ornaments in females have evolved as a result of direct selection from males on more ornamented females (reviewed by Amundsen, 2000), or potentially by female–female competition for mates. Secondly, the alternative “genetic correlation” hypothesis suggests that decoration in mutually ornamented species is adaptive for males and might be neutral or maladaptive for females. Still, females evolve such finery as a consequence of strong benefits of the sexual selection on males and both sexes sharing the genes coding for the ornament (Lande, 1980). A third “social selection” hypothesis advocates that selection for ornaments operates differently in the two sexes, and ecological factors related to female–female competition, not just selection pressures related to mate acquisition, are responsible for shaping ornaments in females (Heinsohn et al., 2005, LeBas, 2006, reviewed by Tobias et al., 2012). Ornaments may be badges of status associated with increased boldness or aggression toward other females (Clutton‐Brock, 2009; Pryke, 2009). In addition to these three hypotheses, female showy traits may evolve simply to advertise readiness to reproduce, or as warning signals (aposematism).

Empirical support for the different hypotheses on evolution of ornaments in females of mutually ornamented species in general are ambiguous (reviewed by Amundsen, 2000, Kraaijeveld et al., 2007, Clutton‐Brock, 2009, Nordeide et al., 2013, Svensson & Wong, 2011, Tobias et al., 2012, see also Lüdtke & Foerster, 2019, Lüdtke & Foerster, 2018, Cotton et al., 2014, Sganga & Greco, 2019, Enbody et al., 2018, LaPlante, 2015, Belliure et al., 2018). The shape of the association between ornaments and fecundity may vary among species and at least part of this variation may affect whether male mate choice of the more showy females is adaptive or not (Watson & Simmons, 2010).

The three‐spined stickleback (*Gasterosteus aculeatus*) is a model species in studies of evolution and behavior in general and in studies of evolution of ornaments (Bell & Foster, 1994; KcKinnon et al., 2019; Wootton, 1976). Male sticklebacks develop blue eyes and a yellow to red—hereafter in this paper referred to as “red” or “reddish”—carotenoid‐based throat prior to the reproductive period (Rowland, 1994). The red throat elicits territorial aggression during the reproductive period (ter Pelkwijk & Tinbergen, 1937; Rowland, 1994; Tinbergen, 1948) and acts as an attractive signal when females choose among males as mates (Milinski & Bakker, 1990; Rowland, 1994). The eyes of sticklebacks have four cone pigments with visual peak absorption maxima around 360, 445, 530, and 605 nm, which are sensitive at the ultra‐violet (UV), short‐, middle‐, and long‐wavelength, respectively (Lythgoe, 1979; Rowe et al., 2004). Female sticklebacks’ sensitivity to red varies annually and is higher during the period of reproduction (Cronly‐Dillon & Sharma, 1968). Males court females more when illuminated by full‐spectrum light including UV, compared to under light lacking UV (Rick & Bakker, 2008a), and especially long (“red”) and short (UV) wavelengths are important when females court males (Rick & Bakker, 2008b). Female and nonreproductive male sticklebacks typically form shoals and feed together with conspecifics both during the reproductive and the nonreproductive part of the year, and dominance relationships may develop within such groups (Bakker, 1994; Peuhkuri et al., 1997; Ranta et al., 1992; Wootton, 1976, 1984).

Contrary to numerous studies on the red throat ornamentation in male three‐spined sticklebacks, studies are few on ornamentation in conspecific females. In short, red throat in females has been reported from North American populations by Bigelow and Schroeder (1953), von Hippel (1999), McKinnon et al. (2000), Yong et al. (2013) and Wright et al. (2015). The pair of pelvic spines is part of the defensive armor protecting three‐spine sticklebacks from gape‐limited predators and have been studied extensively in numerous populations (Gross, 1978; Hagen & Gilbertson, 1972; Klepaker & Østbye, 2008; Moodie, 1972; Rowland, 1994). In North America, only a few studies from California and British Columbia have reported females with red pelvic spines (McKinnon et al., 2000; von Hippel, 1999; Wright et al., 2015; Yong et al., 2013). Red pelvic spines with varying shades of red were reported from both sexes in all the examined 17 populations in North Europe (Amundsen et al., 2015). For a more detailed overview of ornaments in female sticklebacks, see Amundsen et al. (2015).

A few studies have used sticklebacks to test the hypotheses to explain evolution of female ornaments (outlined above). The direct selection hypothesis gained no support in a study where males showed no preference for females with neither red throat or red pelvic spines (Wright et al., 2015), nor in a study where males courted females with red pelvic spines less than dull females (Nordeide, 2002). A negative association between intensity of red pelvic spines and carotenoids content in the eggs, as reported by Nordeide et al. (2006), is not as predicted from the direct selection hypothesis. Yet, the genetic association hypothesis gained some support from a quantitative trait loci (QTL) study reporting a shared genetic architecture coding for the ornamented red throat and pelvic spins in male and female sticklebacks (Yong et al., 2016). Two studies have suggested a very limited role for social selection to explain red ornaments interactions based on controlled experiments of interactions between pairs of live female sticklebacks during their breeding season. In one of the studies, the authors reported lack of a clear link between red throat coloration and female competitive advantage in dyadic experimental trials (Yong et al., 2015). In another study of red showy females from two different populations, neither intensity of red of throat nor of pelvic spines was associated with intraselection aggression (Yong et al., 2018).

Both females and males have red pelvic spines both during and four months after the end of the spawning season in a population in Lake Pallvatnet in North Norway (Amundsen et al., 2015). The redness of the pelvic spines (“Intensity of red” *I_R_*) of sticklebacks from Lake Pallvatnet population (examined in the present study) was intermediate compared to 16 other stickleback populations in Norway. Redness of the pelvic spines in Lake Pallvatnet was higher during the reproductive season although the effect of “season” was moderate especially for females (Amundsen et al., 2015, Figure 5). This suggests confined support to the signaling “readiness to reproduce” hypothesis to explain ornaments in female sticklebacks (see above). We are not aware of any effort to test potential signaling by red pelvic spines in male sticklebacks or nonreproducing female sticklebacks of either sex.

In the present study, we tested the social selection hypothesis by studying whether the intensity of the red color at the sticklebacks’ pelvic spines may act as a badge of status in interactions between nonreproducing conspecifics. The social selection hypothesis includes both social behavioral interactions related to intra‐ and intersexual selection behavior, and nonreproductive behavior to increase survival and food access (Tobias et al., 2012; West‐Eberhard, 1983). The aim of this experiment was to examine behavioral interactions related to ecological factors during the more than 10‐month nonreproductive period as opposed to the sexual behavioral interactions during the reproductive season. Thus, in the rest of the paper we use the term “social selection” to describe interactions during the nonreproductive part of the year, similar to Tobias et al. (2012).

We did this by quantifying behavioral boldness of live sticklebacks toward pairs of dummies, one dummy with (either normal sized or larger than natural) red pelvic spines and one with gray (dull) spines. Unlike the studies by Yong et al. (2015) and Yong et al. (2018), we tested both males and females and we captured the specimens and carried out the experiment during the nonreproductive season. In the present study, the social selection hypothesis would gain support if (i) sticklebacks prefer to interact socially with the dummy with gray pelvic spines at the expense of the dummies with red spines, (ii) a positive association is found between the live sticklebacks’ intensity of red pelvic spines and their preference for the redder of the two dummies.

## 2 MATERIAL AND METHODS

Wild sticklebacks were collected from the 140 m long and 50 m wide and landlocked freshwater Lake Pallvatnet, located at an altitude of 140 m at 67°31’N, 14°40’E in Bodø, North Norway. Fish were caught by traps made of 1.5 L soda bottles deployed along the shore at 0.2–1.0 m depth. The traps fished for 24 hr 9–10 September 2019. Captured sticklebacks were transported to Mørkvedbukta Research Station in Bodø and kept in a 80 L storage tank with continuously flowing water until further handling. The fish in the storage tank were daily fed frozen Chironomidae larvae (Akvarie Teknik, DeLang and Ekman AB, Filipstad, Sweden).

**FIGURE 1:**
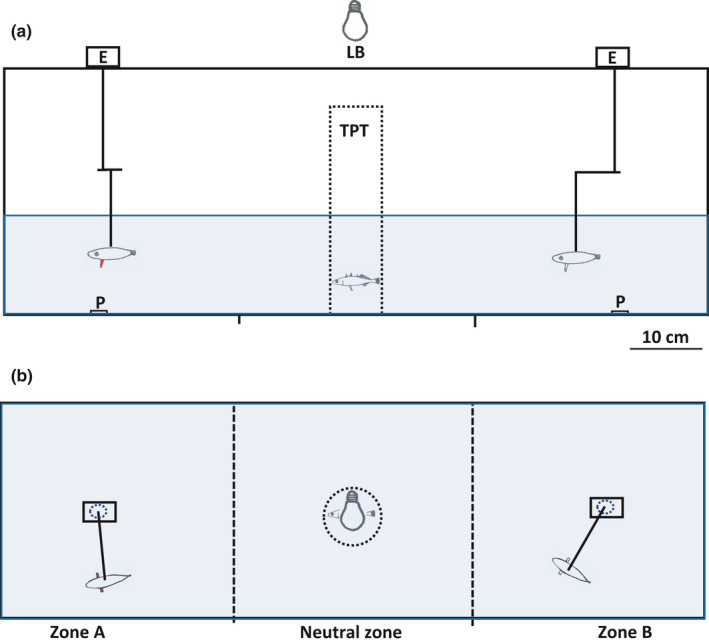
A drawing of the experimental tank from the side (a) and from above (b). Two electric engines (E) moved two dummies in circles at each end of the experimental glass tank. Three sides of the experimental tank were covered by nontransparent plastic foliage. A GoPro camera (not shown) recorded through the fourth uncovered wall, as seen in a). The tank was illuminated by white or green light from a light bulb (LB). TPT, transparent Plexiglas tube; P, petri dishes

The experimental trials were carried out 13 September to 4 November 2019. Trials were run with a total of 37 males and 23 females. Twenty‐four hours before each trial, one focal stickleback was isolated in a transparent Plexiglass tube (7.5 cm in diameter, 35 cm height), hereafter termed the “isolation tube,” and placed inside the storage tank with the other fish. The focal specimen could see its conspecifics in the storage tank and there was a continuous flow of water between the storage tank and the isolation tube. However, the isolated focal stickleback did not have access to food during these 24 hr in order to increase its motivation to forage during the upcoming trials. After 24 hr in the isolation tube, the focal fish was transferred to an experimental tank which consisted of several devices as shown in Figure 1. Two electric engines moved two dummies in circles in each end of the experimental glass tank. The circumference of the circles was 66 cm, and the speed of the dummies was 2.7 cm/s which means that the dummies spent 24 s per lap. Two of the three different dummies (see below) were presented simultaneously in one trial. W.J. Rowland kindly molded the dummies in epoxy in 2003 as described in Rowland (1979). A 52.0 mm nongravid female stickleback was used as model. The dorsal part of the dummies was painted black (85 Coal Black Satin, AAA0655, Humbrol Enamel, Kent, UK) and the ventral part gray (64 Light Grey Matt, AA0713). We mounted artificial pelvic spines on three different dummies. Artificial pelvic spines of approximately normal length (10 mm) were mounted on two dummies, whereas larger (20 mm) artificial pelvic spines were mounted on the third dummy. On one of the dummies with small artificial pelvic spines and the dummy with large pelvic spines, part of the pelvic spines was painted red (60 Scarlet Matt, AAA0655). The red was painted along the entire spine from the base to the tip, covering about 180° of the pelvic spines with the red part directed toward the “body” of the dummies. The remaining part of the pelvic spine on these two dummies was painted with the same gray color as the ventral part of the body of the dummies (see above). The pelvic spines of the remaining third dummy (with normal length spines) were painted gray all over. The artificial spines were installed spread out away from the body. This leaves us with three different dummies: one with gray pelvic spines of normal length (“Normal‐gray” abbreviated “NG”), one with red pelvic spines and normal length (“Normal‐red” abbreviated “NR”), and one with red and large spines (“Large‐red” abbreviated “LR”). Two Chironomidae larvae were presented in each of two Petri dishes below each of the two dummies. The experimental tank was illuminated by either white or green light. The white light came from one light bulb (Anslut, E27 2.5 W 140 lm, rendering average (Ra) of 80, article number 421,433 at www.jula.no), whereas green light came from three bulbs (Anslut, E27 0.7 W 30 lm, article number 420,698). Light intensity was 280 and 120 lux for white and green light, respectively, measured by a light meter (Amprobe LM‐120 Light Meter, Glottental, Germany) inside the experimental tank where the dummies were located. Three sides of the experimental tank were covered by nontransparent green plastic foliage, whereas video recordings were carried out through the fourth uncovered tank wall.

The behavior of each fish was studied under four different combinations of pair of dummies and color of light. The combinations were (i) “Normal‐gray” and “Normal‐red” dummies illuminated by white light, (ii) “Normal‐gray” and “Normal‐red” dummies illuminated by green light, (iii) “Normal‐gray” and “Large‐red” dummies illuminated by white light, and (iv) “Normal‐gray” and “Large‐red” dummies illuminated by green light. Green illumination prevents the sticklebacks from using their red cues which impedes their ability to see red colors (Milinski & Bakker, 1990). Each of the four combinations of dummies and light‐color is hereafter termed a “subtrial”, whereas the combination of the four subtrials with each individual focal fish is termed a “trial.” We randomized both the sequence of the four subtrials with each fish and to which of the two zones (zone “A” or “B,” Figure 1) each of the two dummies appeared in each subtrial.

Five minutes before the actual start of a trial, the focal fish was transferred to the transparent Plexiglas tube (TPT) located inside the experimental tank (Figure 1), in order to become familiar with the two dummies and the tank. A subtrial (the first subtrial of four in a trial) started by removing the Plexiglass tube by pulling a thread while hiding behind a tarpaulin to avoid disturbing the focal fish. The stickleback could then swim freely around in the “Neutral zone” or closer to the two dummies in “Zone A” and “Zone B” and feed freely on Chironomidae larvae beneath any of the dummies (Figure 1). Each subtrial was recorded by a GoPro (San Mateo, US) camera mounted on a tripod in front of the experimental tank. Between subtrials, the focal fish was kept in the isolation tube (in the storage tank) while preparing for the next subtrial with the same focal fish. The trials were carried out in a quiet room. Care was taken not to disturb the fish during the trials, and the focal fish saw no humans from being placed in the TPT tank until the end of the recordings in each subtrial.

After the termination of all four subtrials of a trial, the specimen used in this experiment was killed by an overdose of MS‐222 and frozen in –20°C in darkness awaiting further analysis. Each individual was measured for total length to the nearest mm and weight to the nearest 0.001 g, and the sex was determined by inspection of the gonads. The ventral part of each fish was photographed using an Olympus E‐M10 with a M. Zuiko ED 60 mm 1:2.8 macro lens and a Nissin i40 flash, with its pelvic spines erected and together with a standardized color palette, for later quantification of the redness of the pelvic spines (see below). The water in the experimental tank was removed and replaced with fresh water before a new trial started with another focal stickleback. Intensity of the red color (*I_R_*) of the pelvic spines of each stickleback and the reddish part of the color palette were quantified separately from the digital photos in RGB mode by Adobe photoshop version 13.1. This method has previously been applied to quantify color by several authors (Amundsen et al., 2015; Nordeide, 2002; Nordeide et al., 2006, 2008; Skarstein & Folstad, 1996; Skarstein et al., 2005; Villafuerte & Negro, 1998). In the quantifications, we encircled the pelvic spines and the reddish part of the color palette and estimated the average density values for all three primary colors R, G, B (red, green, and blue) from the pixels enclosed in each of the areas (Villafuerte & Negro, 1998). Intensity of red (I*_R_*) of the two pelvic spines and of the reddish cardboard was calculated as: I*_R_* = R/(R + G + B). The average I*_R_*‐value for both the pelvic spines was used in the analyses, after correcting for differences between photographs using the I*_R_*‐value from the reddish cardboard (see Nordeide et al., 2006 for details). Repeatability of I*_R_* in previous studies has been high (0.99 in Nordeide et al., 2008, see also Nordeide, 2002, and Nordeide et al., 2006).

The first 5 minutes of each subtrial, starting when the focal fish was released from the transparent Plexiglas tube (see Figure 1), were analyzed using the VLC Media Player. The monitor was set in black and white mode during this analysis to reduce potential subjectivity from the analyzer, since this step hindered distinguishing between both white and green light and between the normal‐gray and the normal‐red dummies. A total of 60 trials were carried out of which 9 were removed from the final dataset due to the sticklebacks being either infected by the endo‐parasite *Schistocephalus solidus* (Eucestoda) (3 individuals), or due to technical problems during recording (2 specimens) or opening of the video files of subtrials (4 specimens). This left us with data from all four subtrials from 51 trials, 29 males with total length 55.2 (S.D. ± 4.84) mm and 22 females with total length 55.6 (±7.69 mm), respectively. During the video analyses, we quantified seven different parameters in each subtrial. First, we quantified to which of the two dummies in a pair of dummies (normal‐gray (NG) versus. normal‐red (NR) or normal‐gray (NG) versus large‐red (LR)), each stickleback approached first after being released from the transparent Plexiglas tube (TPT). This preference was defined by which of the zones “A” or “B” containing one of the dummies—the focal fish entered first (see Figure 1). Second, we quantified the time spent in the neutral zone before the focal fish for the first‐time swam into one of the two zones (Zone A or B in Figure 1) containing a dummy. Third, we quantified near which of the two dummies the focal stickleback spent the most time. This was done by noting in which of the zones “A” and “B” the focal fish was located every 15 s during the first five min (a total of 20 observations) after being released to swim freely in the experimental tank. Fourth, we quantified close to which of the dummies the focal specimen preferred to pick its first Chironomidae larvae. Fifth, we quantified the time spent before this first feeding (in the previous point). Finally, we quantified the Intensity of red (*I_R_*) of the sticklebacks’ pelvic spines and the body length of each stickleback.

Most statistics were carried out with IBM SPSS Statistics version 27, whereas Cohen's d and Cohen's h and statistical power were estimated using the pwr package in R version 4.0.2 (R Core Team, 2020). Significance level was set to 0.05, and all p‐values were two‐tailed except the *χ* ^2^ = tests which are one‐tailed (Sokal & Rohlf, 1981). Statistical tests were carried out on females and males separately and additionally on pooled data from both sexes. Exceptions were tests of “First feeding close to a dummy” (see Results, and Table 1 and Figure 4) where we only tested after pooling data from both sexes due to the relatively low sample size (n) and test of “Effect of the intensity of red pelvic spines of the focal sticklebacks” (Figure 5) where the response variable (*I_R_*) differed significantly between the sexes and thus data were not pooled. Binomial tests were used for frequency data. *t* tests or Mann–Whitney *U* tests were used to compare measurement variables (time spent and the intensity of red pelvic spines) relative to the choice between different dummies, after testing data for deviation from normal distribution using the Kolmogorov–Smirnov test. Box–Whiskers plots show 10th and 25th, median value, and 75th, 90th percentiles. A reviewer suggested that we carry out a linear model for each response variable such as “Behavior ~sex + dummy treatment + light treatment + spine intensity + body size.” Our main argument why this is probably not a good idea are as follows: (i) There are two pairs of dummies involved in this experiment: NG–NR and NG–LR. A single multivariate linear model for each response variable would also compare the behavior of the focal fish toward one dummy in one pair of dummies with the behavior toward another dummy in the other pair of dummies. This does not make sense, and it would flaw our results. Additional challenges are: (ii) Which of several models to pick for presentation when five predictors and their interaction terms are involved and a 0‐model being the best model when using an AIC approach. (iii) Collinearity is involved, and (iv) we would have to remove the power analyses.

**TABLE 1:** Counts of sticklebacks which approach each of two dummies in a pair of dummies, when they first left the neutral zone during a subtrial

Light/Sex	Females		Males		Both sexes pooled	
Dummies	NG NR	NG LR	NG NR	NG LR	NG NR	NG LR
White	12 10 (*p* = .832)	10 12 (*p* = .832)	10 19 (*p* = .136)	14 15 (*p* = 1.00)	22 29 (*p* = .401)	24 27 (*p* = .780)
Green	10 12 (*p* = .832)	9 13 (*p* = .523)	16 13 (*p* = .711)	15 14 (*p* = 1.00)	26 25 (*p* = 1.000)	24 27 (*p* = .780)

Note

The sticklebacks were allowed to choose between a pair of dummies with either normal‐sized gray (NG) and normal‐sized red (NR) pelvic spines, or a pair with NG and large red (LR) pelvic spines. P‐values are from the Binomial test comparing the observed number of specimens with the random choice (test proportion = 0.5).

The null hypotheses were not rejected in most of the statistical tests: *t* tests, Mann–Whitney *U* tests and binomial tests, in this study (see Results). Thus, we estimated statistical power (the probability 1 – β of correctly rejecting a false null hypothesis) for some of the tests (see “Results” and “Power analysis” below). Power is a function of effect size, and a larger difference between the two means to be compared in *t* tests, or more specimens preferring one particular of the two zones with the dummies at the expense of the other zone in the binomial tests, would increase the power of our tests. Thus, we iteratively increased the effect size until the statistical tests turned out as marginally significant (*p* < .05), and then, we re‐estimated statistical power using this new effect size. (i) When increasing the effect size in the *t* tests, we kept the sample sizes and standard deviations from actual datasets unchanged. We also kept one of the estimated sample means constant, whereas the other mean was gradually changed until the difference between means became marginally significant (*p* < .05), when tested by *t* test. We then estimated Cohen's d based on this new simulated mean difference and pooled sample standard deviations, in accordance with Cohen (1988). Finally, statistical power was calculated for two‐sided *t* tests, using the pwr.t2n.test of the pwr package, by inserting actual sample sizes, the newly calculated Cohens d and a significance level of 0.05. (ii) Similarly, to estimate statistical power of the binomial tests we gradually increased the difference in number of observations between the two groups while keeping the total sample size (n) constant, until the p‐value from the binomial test turned out to be slightly significant. Then, we used these new counts (which were now significantly different) to estimate Cohen's h and statistical power.

By these simulations, we were able to estimate the magnitude of effect sizes (difference between two means in *t* tests or counts in binomial tests) to detect significant differences between the groups. Effect sizes of Cohen's d and Cohen's h less than 0.5, between 0.5 and 0.8, and above 0.8, are considered as “small,” “medium,” and “large,” respectively (Cohen, 1988). Finally, statistical power was estimated using the new simulated parameters.

The study was carried out in accordance with ethical guidelines stated by the Norwegian Ministry of Agriculture and Food through the Animal Welfare Act. According to these guidelines, we were not supposed to—and therefore do not—have a specific approval or approval number.

## 3 RESULTS

### 3.1 General comments

All focal sticklebacks behaved apparently calmly during the entire five minutes for each of the four subtrials for all the 51 specimens. The fish swam slowly around in the tank or part of the tank, interrupted by periods of varying length where they stayed motionless. We observed no apparent fright of or attraction toward any of the dummies although some focal fish swam close to the dummies. Agonistic behavior or swimming in circles after the dummies was not observed. No burst‐swimming was observed except for a few specimens which burst‐swam when the transparent Plexiglas tube (Figure 1) was removed in the start of the subtrial and the focal fish could start swimming freely in the tank.

#### 3.1.1 First approach toward one of the dummies

Which of the dummies was visited first, when the sticklebacks left the neutral zone for the first time and entered one of the zones containing a dummy, is presented in Table 1. The sticklebacks’ preference between dummies, regardless of the light regime used (e.g., females preference toward the normal‐gray (NG) and normal‐red (NR) dummies illuminated by white light), was not significantly different from random (0.5) for neither females nor males tested separately or pooled.

**FIGURE 2:**
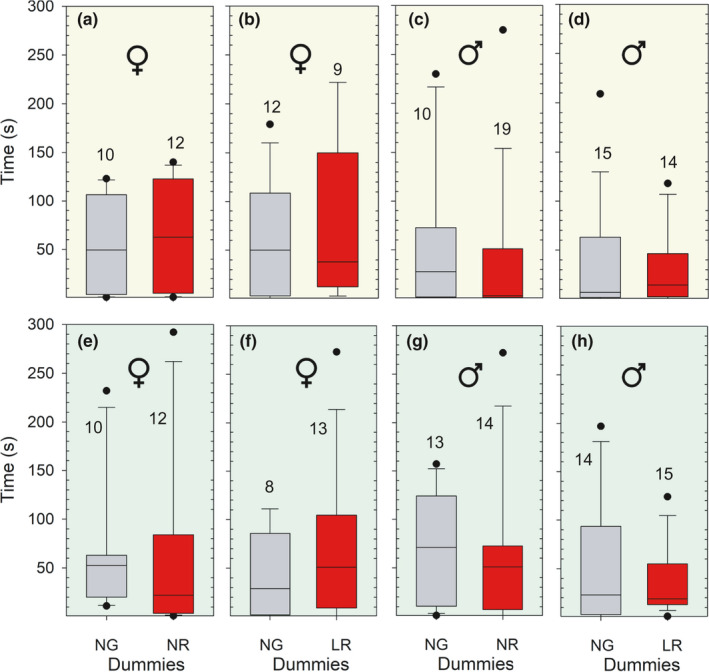
Delay in seconds until released sticklebacks approached one out of two possible dummies in a pair for the first time, illustrated by Box–Whiskers plot. “NG” and “NR” denotes dummies with normal‐sized gray pelvic spines and normal‐sized red pelvic spines, respectively, while “LG” denotes a dummy with large red pelvic spines. The upper four subtrials (a–d) are illuminated with white light, whereas the lower four (e–h) are under green light. Females are shown in a–b and e–f, and males in c–d and g–h. The numbers inside the figures show sample sizes (*n*)

No significant difference was found in the time sticklebacks spent from being released to swim freely in the experimental tank (see Figure 1), and until they left the neutral zone for the first time and swam into one of the zones “A” or “B” (Figure 2a–g). This applied regardless if sticklebacks chose between normal‐gray (NG) and normal‐red (NR) or between normal‐gray (NG) and large‐red (LR) dummies, and regardless of illumination (white or green light). Twelve different tests were carried out, with females only, males only, and females and males pooled. The lowest p‐value found was 0.254 (*U* = 42, *p* = .254, N_1_ = 10 and N_2_ = 12, Mann–Whitney *U* test), and this was when testing females’ preference between normal‐gray (NG) and normal‐red (NR) dummies illuminated by green light (see Figure 2e).

#### 3.1.2 Times observed close to each of the dummies

**FIGURE 3:**
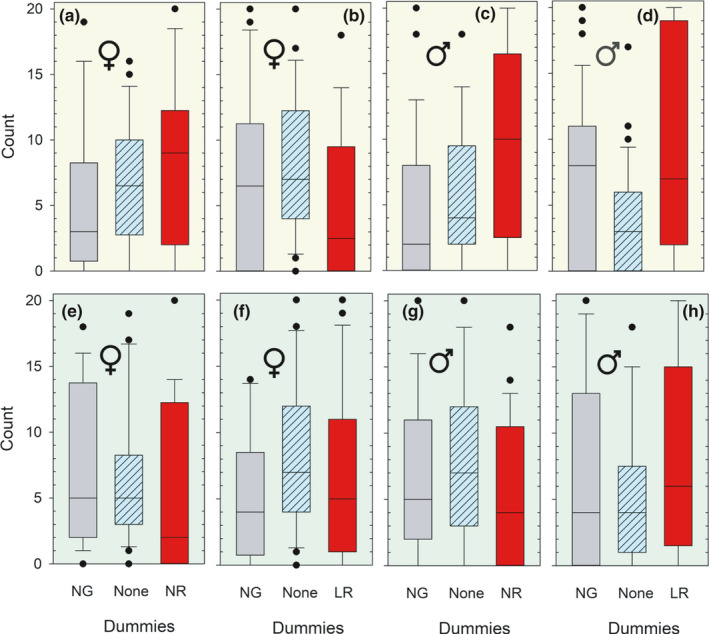
Distribution of the focal sticklebacks in the three zones of the experimental tank, quantified as number of counts per 15 s during 5 min. The x‐axis classes represent the three different zones of the experimental tank. “NG” denotes the zone of the normal‐sized and gray pelvic spine dummy, “NR” the zone of the normal‐sized and red dummy, whereas “LR” is the zone of the large‐sized and red pelvic spined dummy. “None” represents the neutral zone without dummies (see Figure 1). The ordinate shows accumulated no. of registrations in each zone for different lighting (white versus. green, follows the color of the figure) and gender. Females are shown in a–b and e–f, and males in c–d and g–h. The number of different specimens (sample size or N) was 22 for the females and 29 for the males. The data are presented as Box–Whiskers plots

The focal sticklebacks’ positions in the experimental tank—whether in the neutral zone or in one of the zones A or B (near the dummies, see Figure 1)—is presented in Figure 3a–h. Males were observed significantly more often close to the normal‐red (NR) dummy than to the normal‐gray dummy when this pair of dummies was presented to the focal sticklebacks under white light (Figure 3c) (NG compared to NR: *U* = 216.5, *p* = .01, N_1_ = 29, N_2_ = 29, Mann–Whitney *U* test). This difference was significant also after pooling the two sexes (pooling data in Figure 3a and c) (*U* = 784.5, *p* < .01, N_1_ = N_2_ = 51), but not when including only females (Figure 3a) (NG compared to NR: *U* = 177, *p* = .125, N_1_ = N_2_ = 22). None of the other nine tests with females, males, or females and males pooled turned out as significant (*p* > .125).

#### 3.1.3 First feeding close to a dummy

In only 34% (69 of the 204 subtrials) did the sticklebacks feed at least once on the Chironomidae larvae located below any of the two dummies during the first five min. The low feeding incidence gave relatively low sample size (*n*) after splitting the data based on both illumination and on both pairs of dummies, and this applied even after pooling the sexes (Table 2). We found that the frequency of sticklebacks’ choice between the dummies of a given pair did not differ significantly from random (0.5) (Table 2).

**TABLE 2:** Counts of sticklebacks which ate their first piece of food near each of the two dummies in a pair of dummies

Light/Dummies	NG NR	NG LR
White	4 9 (*p* = .267)	7 15 (*p* = .134)
Green	6 8 (*p* = .791)	8 12 (*p* = .503)

Note

The sticklebacks were allowed to choose between eating near a pair of dummies with either normal‐sized gray (NG) and normal‐sized red (NR) pelvic spines, or a pair with NG and large red (LR) pelvic spines. *p*‐values are from the Binomial test comparing the observed number of specimens with the random choice (test proportion = 0.5). Counts are presented after pooling both sexes due to the low numbers.

**FIGURE 4:**
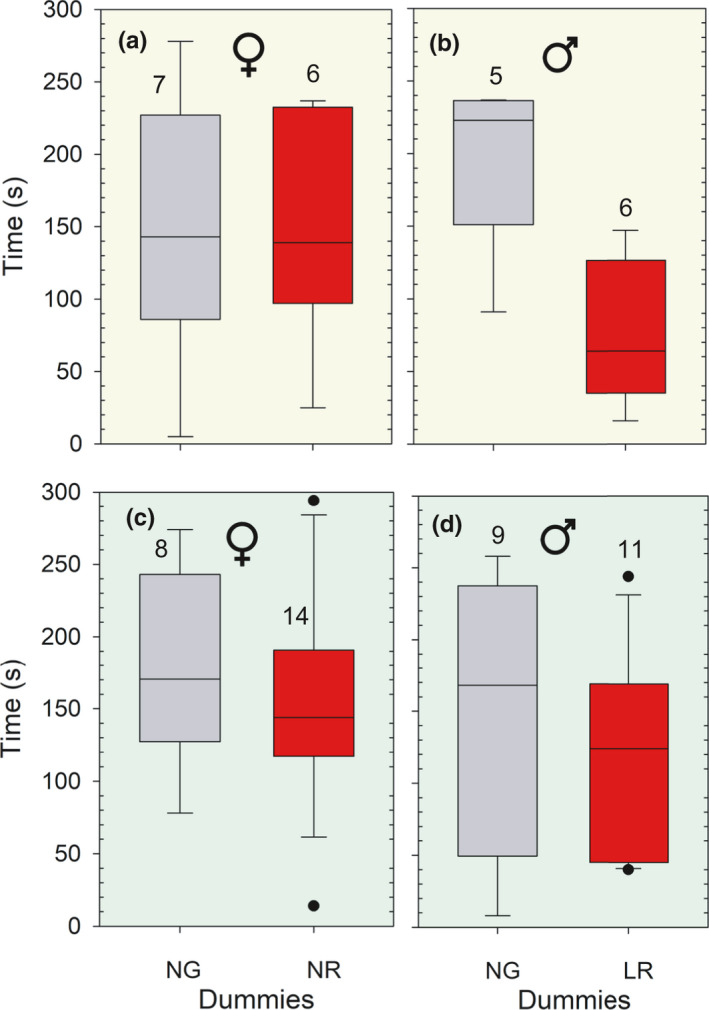
Time spent until sticklebacks ate their first piece of food near each of the dummies in a pair of dummies during a period of 5 min. The dummies had either normal‐sized gray pelvic spines (NG) or normal‐sized and red pelvic spines (NR) in one subtrial, or normal‐sized gray (NR) and large red (LR) pelvic spines. Data are presented by Box–Whiskers plots. Data for both sexes are pooled due to the relatively low total number of observations. Subtrials illuminated with white and green light are shown in a–b and c–d, respectively. Females are shown in Figure 4a and 4c, and males in Figure 4b and 4d. The numbers inside the figures show sample sizes

The time spent before the sticklebacks ate their first piece of food close to one of the dummies is shown in Figure 4a–d. The time males spent before feeding close to the normal‐gray (NG) and the large‐red (LR) when illuminated by white light, differed significantly (*U* = 4, *p* = .014, N_1, 2_ = 5, 9, Mann–Whitney *U* test). The male sticklebacks spent less time before they ate close to the Large‐red (LR) dummy (Figure 4b). No difference was found in time spent for the other subtrials, and the lowest p‐value of the remaining three tests was 0.47 (Binomial test) (Figure 4d).

#### 3.1.4 Effect of the intensity of red pelvic spines of the sticklebacks on which dummy to approach first

**FIGURE 5:**
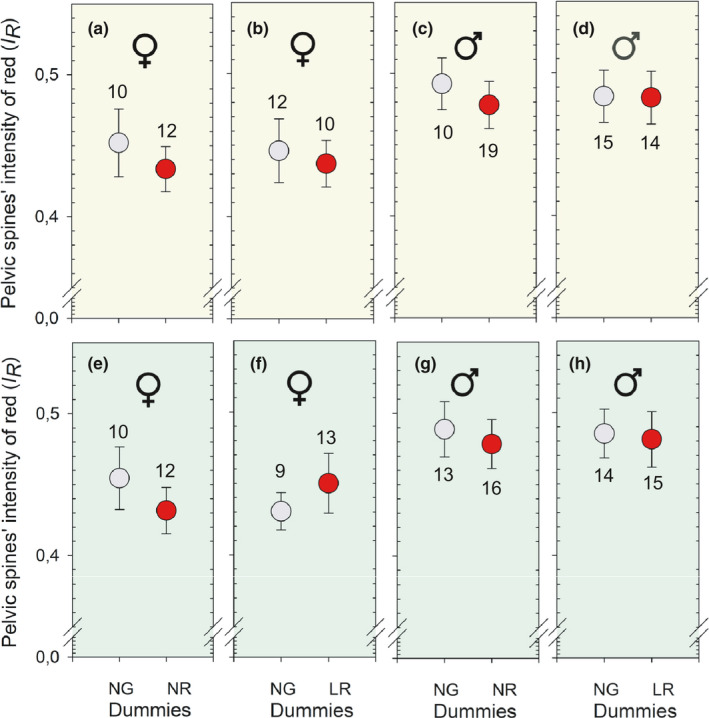
Intensity of red (*I_R_*) at the pelvic spines (mean ±95% confidence interval) of female (5a‐b and Figure 5e–f) and male (Figure 5c–d and Figure 5g–h) sticklebacks relative to which of the dummies in a pair of dummies was approached first. The experiment was carried out when illuminated with white and green light as shown in a–d and e–h, respectively. The numbers inside the figures show sample sizes (*n*)

No effect was found of the Intensity of red (*I_R_*) of the focal sticklebacks on their choice of which dummy in a given pair of dummies in neither females (Figure 5a, b, e, f) or males (Figure 5c, d, g, h). This applies regardless if they were presented to a pair of dummies with normal‐gray (NG) and normal‐red (NR) or a pair with NG and large‐red (LR) spines, and regardless of the illumination by white (Figure 5a‐d) or green (Figure 5e–h) light. The lowest *p*‐value of the eight statistical tests was 0.073 (t = 1.894, *p* = .073, *df* = 20, independent samples *t* test) and appeared when females were illuminated with green light and presented to the normal‐gray (NG) and normal‐red (NR) pair of dummies (Figure 5e).

#### 3.1.5 Effect of body size of the focal sticklebacks on which dummy to approach

**FIGURE 6:**
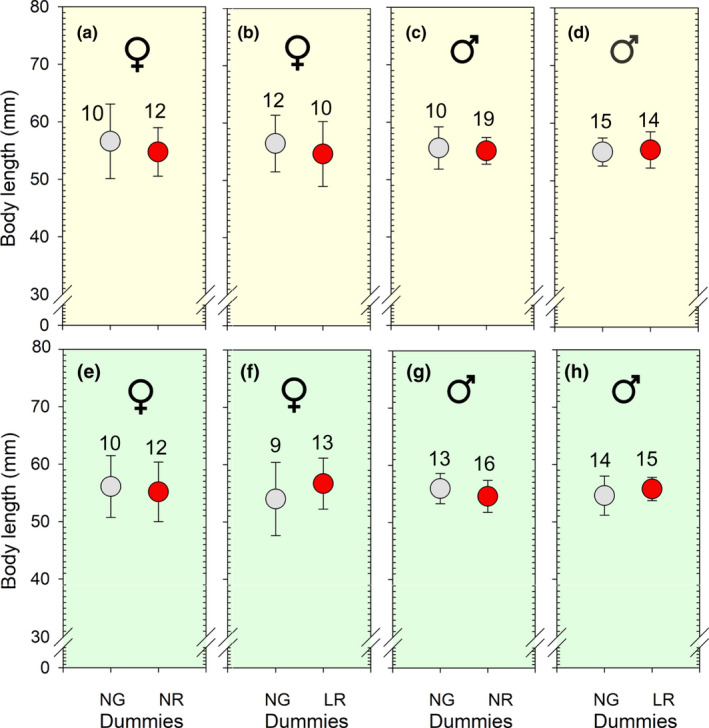
Body length (mean ± 95% confidence interval) of the female (Figure 6a, b and Figure 6e, f) and male (Figure 6c, d and Figure 6g, h) sticklebacks, which approached first one of the dummies in pairs of dummies. The experiment was carried out when illuminated with white and green light as shown in a–b and c–d, respectively. The number above each symbol shows sample size (*n*)

No effect was found of the body length of the focal sticklebacks on their choice of which dummy to approach in a pair of dummies, neither for females (Figure 6a, b, e, f) nor for males (Figure 6c, d, g, h). This applies regardless of which combination of dummies they were presented to, and regardless if the illumination was white (Figure 6a–d) or green (Figure 6e–h). The lowest *p*‐value of the 12 statistical tests was 0.296 (*t* = −1.057, *p* = .296, *df* = 49, independent samples *t*‐test), found for females and males pooled when illuminated with green light and presented to the normal‐gray (NG) and large‐red (LR) pair of dummies (Figure 6f and h pooled).

#### 3.1.6 Power analysis

First, we consider the *t* tests where we compared the intensity of red color (*I_R_*) of the pelvic spines of sticklebacks which approached one or the other of the two dummies in a pair of dummies (Figure 5). For example the mean (*SD*, *n*) *I_R_* of males approaching the normal‐gray (NG) and the normal‐red dummies under white light (Figure 5c) was 0.493 (0.02544, 10) and 0.478 (0.03449, 19), respectively. The difference in *I_R_* between the two groups was not significant (*t* = 1.314, *p* = .200, *df* = 27, independent samples *t* test). Cohen's d was 0.47, and power was 0.21 which are both low. An increase in the larger group mean *I_R_* from 0.493 to 0.502, while keeping the mean of the other group and the standard deviations and sample sizes (*n*) of both groups unchanged, would give a significant difference (*p* = .043) between the means of the two groups. The new Cohen's d and power would increase to 0.76 and 0.46, respectively. Second, Cohen's h was estimated from the actual data of how many of the sticklebacks preferred to approach one of the two dummies of a pair of dummies (see first part of the paragraph above entitled “First approach toward one of the dummies”). After pooling males and females, our data showed that 22 specimens (males and females pooled) first approached the normal‐gray (NG) and 29 first approached the normal‐red (NR) dummies, when they left the neutral zone for the first time (Table 1). Cohen's h was 0.28 and power 0.50 of a binomial test with 22 and 29 specimens preferring each dummy. Thus, both the h and power were low. Changing the outcome to 18 specimens preferring one dummy and 33 preferring the other dummy (from 22 and 29) would give a marginal significant difference (*p* = .049, binomial test). Cohen's h and power calculated based on these significant counts would give values of 0.60 and power of 0.99, respectively. Cohen's d‐ or h‐values between 0.5 and 0.8 and above 0.8 are classified as medium‐ and large‐sized differences, respectively, according to Cohen (1988). To sum up these power analyses, our design showed low power to detect small effect sizes (<0.5) according to Cohen (1988), but showed sufficient power to detect effects in the upper middle (0.5–0.8) and high range (>0.8).

#### 3.1.7 Testing the experimental setup

**FIGURE 7:**
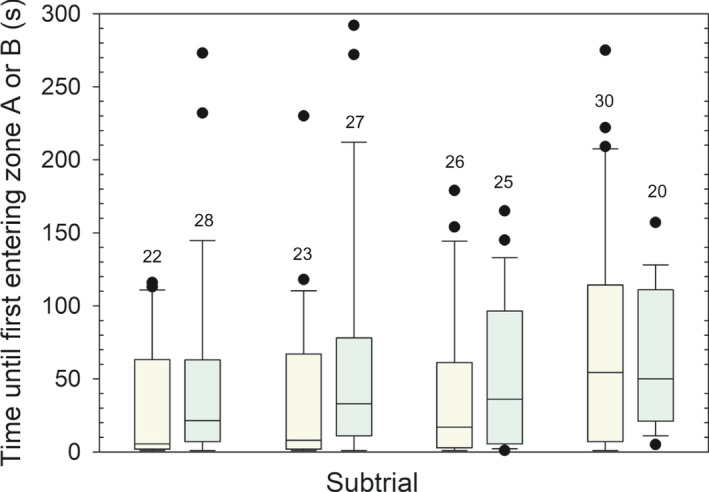
Time spent by sticklebacks until they entered a zone of any dummy. The x‐axis numbers denote the temporal sequence of the four subtrials for a focal fish. Subtrials illuminated with white and green light are shown in yellow and green, respectively. The data are presented as Box–Whiskers plots and the number above each box plot show sample size (*n*). Data for both sexes are pooled

Our results suggest a nonsignificant trend of increased time spent until first entering one of the zones near the dummies A or B, following the exposure of sticklebacks to an increasing number of subtrials, (*χ* ^2^ = 6.556, *p* = .087, *N* = 51, Friedman test) (Figure 7). Concerning the same behavioral parameter, the sticklebacks did not differ significantly in time spent under white compared to green light in any of the first (*U* = 253.0, *p* = .281, N_white_ = 22, N_green_ = 28, Mann–Whitney *U* test), second (*U* = 221.5, *p* = .082, N_white_ = 23, N_green_ = 27), third (*U* = 277.5, *p* = .370, N_white_ = 26, N_green_ = 25), or fourth subtrial (*U* = 272.0, *p* = .790, N_white_ = 30, N_green_ = 19) (Figure 7). Moreover, of the 69 (of 204) subtrials where the sticklebacks actually fed on a piece of Chironomidae larvae, 42 and 27 of these subtrials were illuminated by green and white light, respectively. This difference did not differ significantly from random (50%) (*p* = .091, binomial test). To test for symmetry in the experimental tank, we counted number of subtrials when the focal fish entered zone “A” and “B” after leaving the neutral zone for the first time, regardless of which dummy was present in which zone. The result was 118 and 86 subtrials in zone “A” and “B,” respectively, and this difference was significant (*χ* ^2^ = 5.0196, *p* < .05, *df* = 1, chi‐square test). Repeating the same test but including only the first of the four subtrials for each fish, the numbers were 25 and 26 for zone “A” and “B,” respectively, and these numbers were not significantly different (*χ* ^2^ = 5.0196, *p* > .05, *df* = 1).

## 4 DISCUSSION

Three of the 68 statistical tests carried out turned out as significant, which is as expected from random events at a 0.05 significance level. In addition to the lack of any nonsignificant trend in the results and the estimated power of the statistical tests, this suggests no support for either (i) sticklebacks behaved differently toward one or the other of the dummies or (ii) that the intensity of red of the focal sticklebacks’ pelvic spines or their body length affected their behavior toward the dummies.

The results from the present study was carried out several months after the end of the reproductive period. The results conform to results from similar experimental behavioral studies of female sticklebacks with red carotenoids‐based (throat or spine) ornaments carried out during the reproductive period as outlined in the Introduction (Yong et al., 2015, 2018). Thus, the present study concurs to the lack of support to the social selection hypothesis to explain evolution of red ornaments in female sticklebacks. Concerning male sticklebacks, our results suggest that males are ignorant toward red versus gray color of their conspecifics’ pelvic spines outside the spawning period. This is in contrast to studies carried out during the spawning period, when male sticklebacks behave aggressively toward other males with a red ornamented throat and toward nongravid females conspecifics which enter their territory (Rowland et al., 1995; Tinbergen, 1948; Wootton, 1976).

The result from this study adds to the studies which so far all have failed to demonstrate any functional significance of the red pelvic spines for neither male nor female sticklebacks during the nonreproductive season (this study), or for female three‐spined sticklebacks during the reproductive season (see Introduction). A nonadaptive function of the red spines does not support either the social selection hypothesis or the direct selection hypothesis, whereas it may concur with the genetic correlation hypothesis which is the third of three main hypotheses presented in the Introduction. However, this requires that red pelvic spines give some advantages for the males during at least part of the year. On such potential advantageous effect would be that males with elaborately colored red pelvic spines have an advantage during reproduction. This might well be the case since red color at the males’ throat is advantageous during aggressive male–male interactions during the reproductive season and is an attractive signal when females chose among males as mates (see Introduction). If future studies demonstrate that also red pelvic spines, and not only red throat, increase males fitness during the spawning season, this might explain the presence of red pelvic spines in males. If so, this might also explain the red spines in females according to the genetic correlation hypothesis, since males and females share most of the genome. However, it remains to demonstrate that male sticklebacks have fitness advantages of red pelvic spines. Otherwise, lack of any adaptive value of the red pelvic spines what so ever would concur with nonadaptionist arguments by Caro (2021) and Gould and Levontin (1979).

Carrying out controlled behavioral experiments involves challenges. In the present experiment, we introduced the wild fish to stimuli—artificial dummies—which they had never encountered before. On the other hand, studies of sticklebacks’ behavioral toward artificial dummies have been carried out (by authors) for more than 80 years (Bolyard & Rowland, 1996; ter Pelkwijk & Tinbergen, 1937; Rowland, 1982, 1989, 1994; Tinbergen, 1948). However, other authors have advocated that both individuals and populations vary in their responsiveness to dummies (Rowland & Sevenster, 1985; Wootton, 1971). Concerning illuminating the experimental tank with green light, previous studies on sticklebacks have suggested that the color of the light does matter when it comes to mate choice involving choosing between two conspecifics of the other sex which differ in redness (Milinski & Bakker, 1990; Nordeide, 2002). Yet, the actual activity of the courtship was not affected by green versus white light in these studies (Milinski & Bakker, 1990; Nordeide, 2002). This concurs with the present study where the time spent for the sticklebacks to leave the neutral zone and swim into one of the zones containing a dummy did not differ significantly under white versus green light. We have no explanation to the observed lack of symmetry in the sticklebacks’ choice of dummy to approach when leaving the neutral zone for the first time when including all subtrials, but not when including only the first of the four subtrials. Our use of the computer monitor's black and white mode during the video analyses reduced potential subjectivity during the videos analyses. This applies both to the quantification of the sticklebacks’ behavior toward one or the other of the normal‐gray or normal‐red pair of dummies and to quantification of the sticklebacks’ behavior toward dummies under white and green light. The black and white monitor mode did probably not completely eliminate potential bias from the analyzer based on the different size of the normal‐gray and large‐red dummies’ pelvic spines. Another potential challenge is that each specimen was studied in four consecutive subtrials which may potentially cause the focal sticklebacks to learn or habituate to the dummies and the experimental setup. However, no significant effect of learning or habituation was revealed. Moreover, each focal fish in this study was tested only once toward a specific pair of dummies when illuminated by a specific color (white or green). Thus, this experiment was carried out without replicates. Each focal specimen was tested four times (four “subtrials”). If we had carried out replicated subtrials, this number would increase from four to eight. We chose not to do the eight subtrials because this high number of subtrials might lead to learning (or habituation) which would again flaw the results. When we tested for potential effect of learning after four subtrials, we revealed a slight and insignificant increase in time until the focal fish entered one of the zones with a dummy (presented in Figure 7). Thus, to overcome the potential problem with habituation we could either (i) risk flawing the entire experiment by exposing each focal specimen to too many subtrials leading to habituation, (ii) reduce the number of dummies presented from three to two by for example removing the dummy with large red spines which would reduce the number of pairs of dummies from two to one, or (iii) run the experiment without replicates. We decided to go for the latter option. Finally, we cannot know for sure whether the male or female sticklebacks regarded the dummies as specimens of their own or of the opposite sex, even though the dummies were molded from a female stickleback (see Material and methods). On the other hand, the sex of conspecific competitors might be of minor concern during periods of the year when they do not reproduce.

To conclude, the intensity of red at the pelvic spines and the size of the pelvic spines seem not to influence any of the measurements of the sticklebacks’ behavior toward dummies. Behavior toward the dummies was not associated with intensity of red at the pelvic spines of the focal sticklebacks or their body length. Thus, this study does not support predictions from the social selection hypothesis to explain evolution of red pelvic spines in three‐spined sticklebacks.

## CONFLICT OF INTEREST

None declared.

## AUTHOR CONTRIBUTION

**Karl Kristian Kroken:** Conceptualization (supporting); Data curation (lead); Formal analysis (equal); Funding acquisition (supporting); Investigation (equal); Methodology (equal); Project administration (supporting); Resources (equal); Software (supporting); Supervision (supporting); Validation (supporting); Visualization (supporting); Writing‐original draft (supporting); Writing‐review & editing (supporting). **Axel Aas Sæthre:** Conceptualization (supporting); Data curation (lead); Formal analysis (equal); Funding acquisition (supporting); Investigation (equal); Methodology (equal); Project administration (supporting); Resources (equal); Software (supporting); Supervision (supporting); Validation (equal); Visualization (supporting); Writing‐original draft (supporting); Writing‐review & editing (supporting). **Ove Nicolaisen:** Conceptualization (equal); Data curation (supporting); Formal analysis (equal); Funding acquisition (supporting); Investigation (equal); Methodology (equal); Project administration (supporting); Resources (supporting); Software (equal); Supervision (equal); Validation (equal); Visualization (equal); Writing‐original draft (equal); Writing‐review & editing (equal). **Torvald Blikra Egeland:** Conceptualization (equal); Data curation (supporting); Formal analysis (equal); Funding acquisition (supporting); Investigation (equal); Methodology (supporting); Project administration (supporting); Resources (supporting); Software (equal); Supervision (equal); Validation (equal); Visualization (supporting); Writing‐original draft (equal); Writing‐review & editing (equal). **Jarle Tryti Nordeide:** Conceptualization (lead); Data curation (supporting); Formal analysis (equal); Funding acquisition (lead); Investigation (equal); Methodology (lead); Project administration (lead); Resources (lead); Software (supporting); Supervision (lead); Validation (equal); Visualization (equal); Writing‐original draft (lead); Writing‐review & editing (lead).

## Data Availability

The data presented in this study are archived at Dryad https://doi.org/10.5061/dryad.1rn8pk0th
